# Complete Favorable Response after Second-Line Immunotherapy in Stage IV Non-Small Lung Cancer with Visceral Metastases and Operated Brain Metastasis

**DOI:** 10.3390/jpm14070754

**Published:** 2024-07-16

**Authors:** Roxana-Andreea Rahnea-Nita, Laura-Florentina Rebegea, Radu-Valeriu Toma, Alexandru Nechifor, Georgiana Bianca Constantin, Gabriela Rahnea-Nita

**Affiliations:** 1 Clinical Department, Faculty of Medicine, University of Medicine and Pharmacy “Carol Davila”, 050474 Bucharest, Romania; roxana.rahnea-nita@umfcd.ro (R.-A.R.-N.); radu.toma@umfcd.ro (R.-V.T.); 2 Oncology-Palliative Care Department, “Sf. Luca” Chronic Diseases Hospital, 041915 Bucharest, Romania; gabriela.rahnea-nita@umfcd.ro; 3 Clinical Department, Faculty of Medicine and Pharmacy, “Dunarea de Jos” University, 800008 Galati, Romania; laura.rebegea@ugal.ro (L.-F.R.); alexandru.nechifor@ugal.ro (A.N.); 4 Radiotherapy Department, “Sf. Ap. Andrei” County Emergency Clinical Hospital, 800579 Galati, Romania; 5 The Radiotherapy Department, Oncological Institute “Prof. Dr. Alexandru Trestioreanu”, 022328 Bucharest, Romania; 6 Morphological and Functional Sciences Department, Faculty of Medicine and Pharmacy, “Dunarea de Jos” University, 800008 Galati, Romania; 7 Clinical Department, Faculty of Midwifery and Nursing, University of Medicine and Pharmacy “Carol Davila”, 050474 Bucharest, Romania

**Keywords:** non-small cell lung cancer, brain metastasis, radiotherapy, immunotherapy, complete favorable response

## Abstract

Introduction: Patients with non-small cell lung cancer (NSCLC) and brain metastatic disease have an unfavorable prognosis. The goal of the treatment in stage IV NSCLC is to increase the survival rate and to improve the quality of life. Case report: We present the case of a young male patient (47 years old at the time of diagnosis in 2021) with NSCLC stage IV with the onset of the disease through neurological symptoms determined by brain metastasis. The immunohistochemical result raised problems of differential diagnosis. The complete favorable response was obtained 20 months after the initiation of second-line immunotherapy, maintaining this response 6 months later. Discussion: The therapeutic approach for the second-line treatment of patients with metastatic non-small cell lung cancer (NSCLC) without mutations has been revolutionized by the approval of immune-checkpoint inhibitors (ICIs). The combination of radiotherapy and immunotherapy can increase the abscopal phenomenon by the stimulation of an immune response against tumors at distant sites, outside the radiation field, as recent studies suggest. Conclusions: Second-line immunotherapy is beneficial to the survival of patients with NSCLC with disease progression beyond initial chemotherapy. The combination of radiotherapy and immunotherapy has the potential to play an important role in metastatic NSCLC.

## 1. Introduction

Lung cancer represents almost 12% of all cancers in the world [1]. Non-small cell lung cancer (NSCLC) is the predominant form of lung cancer, representing 80% of all lung cancer cases. Histopathologically, NSCLCs are classified into adenocarcinoma, squamous cell carcinoma, large cell carcinoma (LCLC) and adenosquamous carcinoma. The World Health Organization (WHO) classification from 2015 of lung cancer brought the following changes into the classification of lung tumors: important changes in lung adenocarcinoma, the reclassification of squamous carcinoma in keratinizing, non-keratinizing and basaloid subtypes, the restriction of the diagnosis of LCLC, the grouping of neuroendocrine tumors in the same category, a current point of view on histological grading of lung cancer, etc. [2]. The 2021 WHO classification system for thoracic tumors (including lung cancer) contains several updates to the 2015 edition. In 2015, guidelines recommended the evaluation of only two molecular markers: epidermal growth factor receptor (*EGFR*) mutations and anaplastic lymphoma kinase (*ALK*) fusions, in patients with non-squamous non-small cell lung cancer. In 2021, the guidelines recommended the testing of numerous genetic mutations for which targeted therapies are available: *ROS1*, *RET*, *NTRK1–3*, *KRAS*, *BRAF* and *MET* [3,4]. The goal of the treatment in stage IV NSCLC is to increase the survival and to provide a better quality of life. Immune-checkpoint inhibitors (ICIs) play a crucial role in the treatment of advanced non-small cell lung cancer (NSCLC), having the role of prolonging life by stimulating the body’s immune mechanisms, which have the ability to attack cancer cells [5]. Second-line immunotherapy is the standard of care (if not used previously) for patients with NSCLC. The response to immunotherapy as second-line treatment has been demonstrated by the improvement in Overall Survival (OS) and Progression-Free Survival (PFS) [1]. Patients with NSCLC and brain metastatic disease have an unfavorable prognosis, and complete remission in stage IV NSCLC is not common [5,6]. In this article, we report the case of a patient with stage IV non-small cell lung cancer with operated brain metastasis and adrenal metastases, who achieved a complete response with immunotherapy in a second-line setting after the failure of chemotherapy.

## 2. Case Report

We present the case of a 47-year-old (at the time of diagnosis, in 2021) male patient.

The patient worked as a turner and in the asphalt industry; he is currently retired because of illness. The patient was a smoker, consuming20 cigarettes/day from the age of 17 and, from the age of 44, 40 cigarettes/day. He quit smoking in 2021 after he became sick.

The patient presented in May 2021 to the Neurosurgery Clinic of the Bagdasar-Arseni Emergency Clinical Hospital for right brachial paresis resulting from a recent cranio-cerebral trauma. Computed Tomography (CT) and Magnetic Resonance Imaging (MRI) of the skull highlighted a replacement process of the left parieto-occipital space with perilesional edema, measuring 30/30 mm.

Surgery was performed, practicing the quasi-total ablation of the left parietal brain tumor.

A chest, abdomen and pelvis CT performed after the neurosurgical intervention, at the end of May 2021, showed a left apical tumor of 20/12 mm and left lateral-aortic mediastinal lymph nodes.

Thoracic surgery exam was performed in May 2021. A pulmonary resection was recommended after oncological evaluation and cerebral radiotherapy.

The result of the histopathological examination, from the end of May 2021, highlighted the cerebral metastasis of poorly differentiated carcinoma that is frequently associated with clear cells, and immunohistochemical tests were further recommended.

In June 2021, the patient presented to the Oncology-Palliative Care Department of “Sf. Luca” Hospital for chronic diseases from Bucharest. After the general evaluation, the radiotherapy consultation recommended the following: external cerebral radiotherapy, medical oncology consultation in order to undergo a systemic treatment, thoracic surgery consultation for assessing the feasibility of surgical intervention for a stage IV left lung neoplasm with brain metastases (M1BRA) operated.

Between 14 June and 2 July 2021, external radiotherapy was administered with the Volumetric Modulated Arc Therapy (VMAT) technique up to a total dose = 30 Gy (10 fractions per target volume of the whole brain) followed by BOOST up to a total dose of 10 Gy (5 fractions) per target volume former tumor bed.

The immunohistochemical result (IHC), carried out in June 2021, highlighted the following: CK 7 zonal positive; CK 20 negative; TTF1 negative; CD 10 positive on extended areas of the tumor; positive zonal VIM; P 63 negative; CDX 2 inconclusive; KI 67 positive in 20%. The conclusion was that the histochemical examination is most likely compatible with a carcinoma metastasis of renal origin, having a high histological aggressiveness.

Abdominal MRI performed in July 2021 highlights an adrenal nodular lesion on the right side with a most likely secondary appearance.

**Figure 1:**
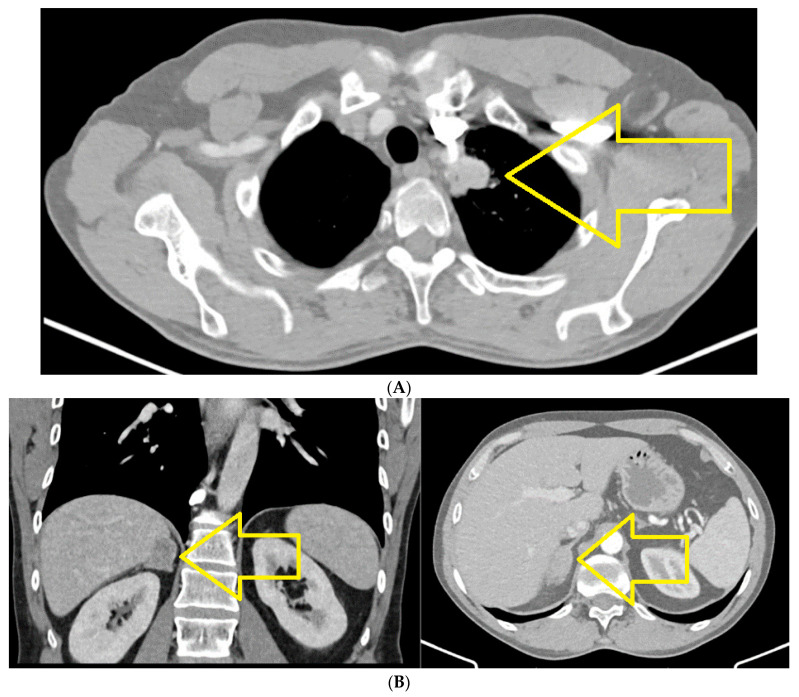
(**A**) Chest CT August 2021: left upper lobe lung tumor. (**B**) CT abdomen August 2021: right adrenal node newly appeared (secondary).

CT of chest, abdomen and pelvis from August 2021 highlights the left upper lobe lung tumor, which was discretely increased in size compared to the previous examination; a right adrenal node also newly appeared was identified (secondary) (Figure 1A,B).

The immunohistochemical examination was reevaluated and the result, from the end of August 2021, highlights the following: VIM negative in the tumor, positive in the stroma (highly excludes adrenal and renal origin; AE1-AE3 positive diffuse in the tumor; MELAN A negative (excludes with a high probability the adrenal origin); SYN negative (rules out the tumor’s zonal origin); CK 7 positive in the tumor cells (suggests an origin in a respiratory epithelium squamous metaplasia); Napsin A negative.

Testing PD-L1: PD-L1 was tested immunohistochemically with DAKO Clone 22C3. There was a positive result in approximately 5% of tumor cells with circumferential membrane reaction, predominantly incomplete, of moderate and high intensity.

Testing ALK: ALK testing (clone D5F3 Ventana, Automat Benchmark) gave a negative result in tumor cells.

EGFR Testing: It was performed by Real-Time Polymerase Chain Reaction (Real-Time PCR). Tissue sections from the formalin-fixed and paraffin-embedded tissue were analyzed, and relevant regions with 50% tumor cell content were selected. DNA was extracted from the selected region. The sample was analyzed for somatic mutations in exons 18, 19, 20 and 21 of the EGFR gene. Result: No mutation was identified in exons 18, 19, 20 or 21 of the EGFR gene.

The conclusion was brain metastasis of poorly differentiated carcinoma with large cells. We were not able to differentiate between a poorly differentiated carcinoma of the respiratory type with squamous metaplasia and a poorly differentiated lung carcinoma differentiated with large cells; thus, correlation with the clinical-imaging data was necessary. PD-L 1 5%, ALK negative, EGFR negative.

Corroborating the immunohistochemical data with the clinical and imaging data, we established the diagnosis: bronchopulmonary neoplasm poorly differentiated NSCLC (poorly differentiated non-keratinizing squamous carcinoma) stage IV; brain metastasis operated, irradiated; right adrenal metastasis.

Cranial CT performed in December 2021 highlights a postoperative left parietal subcortical cavity with axial diameters of 34/18 mm with fine native hyperdense peripheral lyserium.

According to the recommendation of the Multidisciplinary Oncological Commission, from August 2021, first-line chemotherapy with Paclitaxel plus Carboplatin was initiated. In September 2021, the same commission recommended the continuation of chemotherapy with Paclitaxel plus Carboplatin and then re-evaluation after six series of treatment.

**Figure 2:**
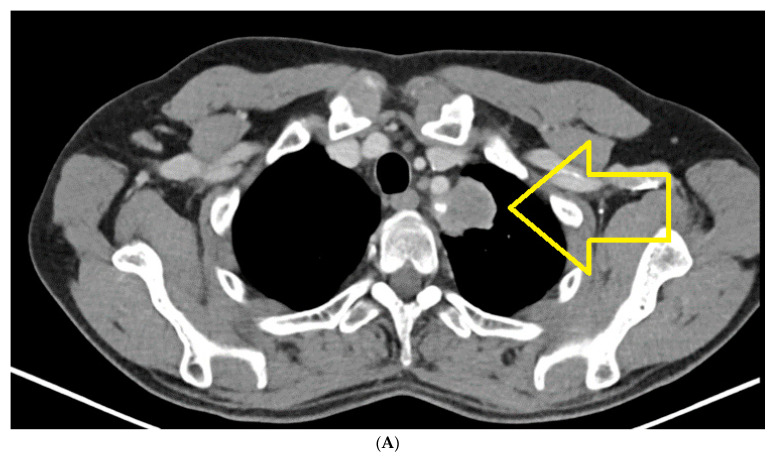

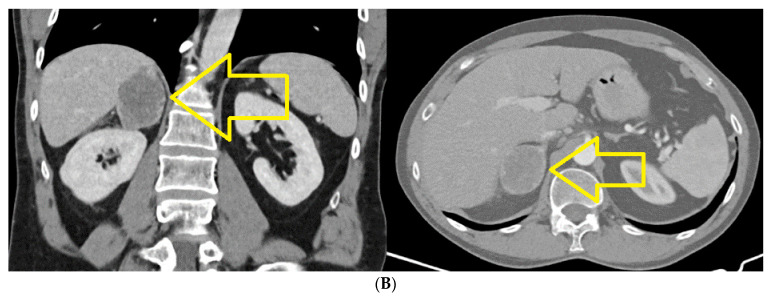
(**A**) Chest CT January 2022: Left upper lobe pulmonary tumor mass in dimensional progression. Small mediastino-hilar lymph nodes, not significantly changed, compared to the previous examination. (**B**) CT abdomen January 2022: Right adrenal secondary determination, in clear dimensional progression, newly appeared left adrenal secondary determination.

CT of chest, abdomen and pelvis from January 2022, compared to CT from August 2021, highlights the following: left upper lobe lung tumor mass in dimensional progression; small mediastino-hilar lymph nodes, not significantly changed, compared to the previous examination; secondary determination of the right adrenal, in clear dimensional progression; secondary determination of the left adrenal newly appeared; no focused trials for lung, liver and bone secondary determinations (Figure 2A,B).

From February 2022, immunotherapy with Nivolumab 240 mg every 2 weeks was initiated, according to the recommendation of the Multidisciplinary Oncological Commission, which is a treatment that the patient is still following at the time of writing this article (May 2024).

CT skull performed in May 2022 highlights the left parietooccipital sequelae, which was apparently unchanged compared to the examination in December 2021.

**Figure 3:**
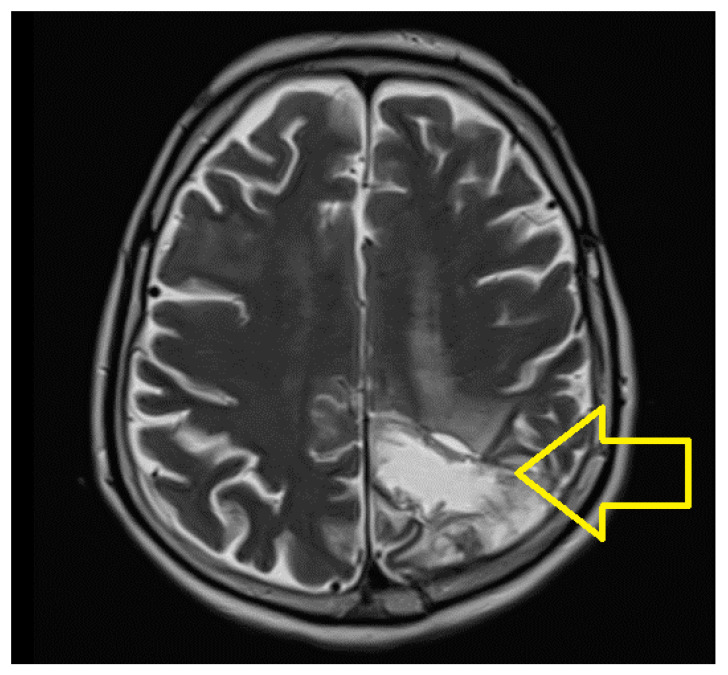
Skull MRI October 2022: left parietooccipital sequela.

A control skull CT was performed in August 2022, and a control skull MRI was performed in October 2022, which did not reveal any other changes (Figure 3).

CT of the chest, abdomen and pelvis performed in December 2022 shows significant dimensional regression (almost complete) of the left lung tumor formation and significant dimensional regression of the secondary right adrenal tumor.

**Figure 4:**
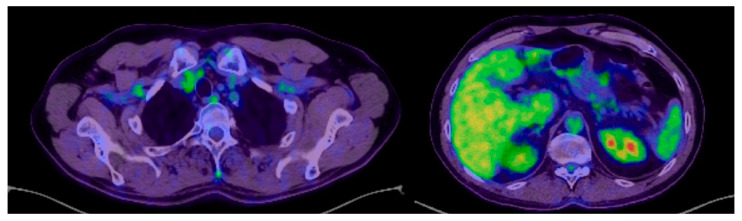
PET-CT October 2023: normal post-therapeutic complete response, without recurrence or metastases.

**Figure 5:**
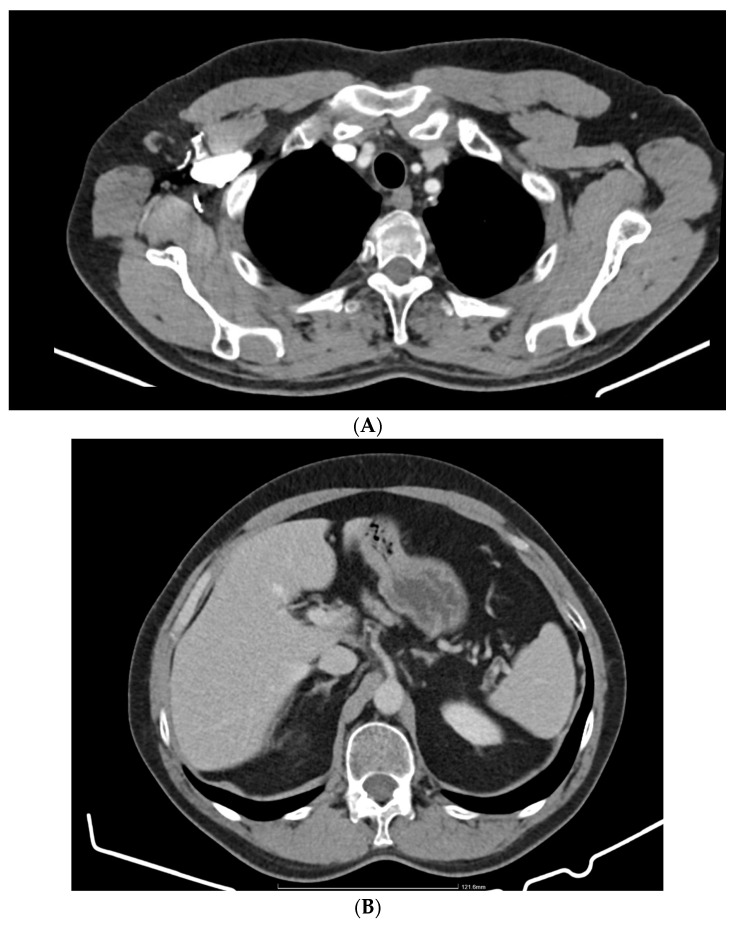
(**A**) March 2024 chest CT: normal appearance. (**B**) CT abdomen March 2024: normal appearance.

The PET-CT performed in October 2023 shows a suggestive aspect for a complete normal post-therapeutic response without relapse or metastases (Figure 4), and the CT of the head, chest, abdomen and pelvis performed in March 2024 shows a normal appearance without relapse or metastases (Figure 5A,B).

Thus, we find a complete favorable response to immunotherapy 20 months after the initiation of treatment with Nivolumab, and this response was maintained 6 months later in March 2024. The treatment was well tolerated without adverse reactions.

In May 2024, 3 years after the diagnosis, the patient has an ECOG performance status = 0, having a very good quality of life. At the same time, the patient was monitored during this period of time, in terms of the presence of symptoms, using the Edmonton Symptom Assessment System (ESAS), and in terms of anxiety and depression, using the Hospital Anxiety and Depression Scale (HADS) [7,8,9,10,11,12,13]. He did not show or communicate any symptoms in those 3 years.

## 3. Discussion

We performed a quick review of the specialized literature, regarding the histopathological types of NSCLC, with an emphasis on the form of large cell lung cancer (LCLC), considering that the immunohistochemical results raised problems of differential diagnosis (brain metastasis of poorly differentiated carcinoma with renal origin/cerebral metastasis of poorly differentiated carcinoma with large cells originating in a respiratory type epithelium with squamous metaplasia/cerebral metastasis of poorly differentiated carcinoma with large cells starting from poorly differentiated lung carcinoma with large cells).

In recent history, large cell lung carcinoma (LCLC) represented one form of non-small cell lung cancer that was found in 9% of all non-small cell lung cancers (NSCLCs), being in 3rd place after adenocarcinoma and squamous cell carcinoma.

This type of cancer has poor differentiation and an unfavorable prognosis because it tends to grow faster and spread easier than other forms of non-small cell lung cancers [14].

Recently, LCLC has become one of the rarest subtypes of NSCLC, since most cases of LCLC are immunophenotypically similar to adenocarcinoma or squamous cell carcinoma [15]. Thus, the diagnosis of LCLC is a diagnosis of exclusion, being an undifferentiated NSCLC, which does not have histological and immunohistochemical aspects of adenocarcinoma, squamous cell carcinoma, or small cell carcinoma and which represents only 1–2% of NSCLC.

Based on 2015 WHO classification criteria in redefining large cell lung carcinoma, the expression of specific markers (TTF1, Napsin A, P 40, CK5/6, CK, vimentin and ZEB1) is analyzed by immunohistochemistry. According to this new classification, most large cell lung cancers have been reclassified into adenocarcinoma and non-keratinizing squamous cell carcinoma [16,17].

LCLC is one of the pathological types of NSCLC, lacking morphological or IHC evidence of adenocarcinoma or squamous carcinoma.

According to the WHO 2015 classification, LCLC no longer includes the three distinct histological entities: large cell neuroendocrine carcinoma (LCNEC), basaloid carcinoma and lymphoepithelioma-like carcinoma (LELC).

The distinction between LCLC and LCNEC is based on the presence of neuroendocrine morphology and the expression of at least one neuroendocrine marker in at least 10% of tumor cells. LCLC with neuroendocrine morphology and without neuroendocrine marker expression is classified as LCC, and it is named LCNEC-null [18,19].

At the same time, the other two morphological entities of LCLC, clear cell and rhabdoid phenotype, are no longer considered subtypes of LCLC, taking into account that they are also found in other types of lung cancer [2,20,21].

Clear-cell carcinoma (histologically composed of large cells with a clear cytoplasm rich in glycogen) should not be considered a distinct clinicopathological entity; clear cells are found both in LCLC and in adenocarcinoma and squamous carcinoma [20,21,22]. Tumors composed even predominantly of clear cells should be classified according to the major WHO categories [20].

Squamous cell carcinoma (SCC) is more strongly associated with smoking than any other type of NSCLC.

SCC of the lungs originates from the transformation of the squamous cells lining the airways.

From a histopathological point of view, aspects such as keratinization or intracellular bridges appear in at least 10% of the tumor mass. If the squamous differentiation is minimal, then the diagnosis is poorly differentiated SCC. From an immunohistochemical point of view, the expression of p63 and p40 biomarkers appears in SCC [23].

Analyzing the immunohistochemical results, together with the data from the literature, we considered that the histopathological type of the patient in our study is poorly differentiated non-keratinizing squamous carcinoma.

Approximately 50% of NSCLC patients develop brain metastases, the prognosis being unfavorable [23].

Aoki et al. [5] conducted a study of 1699 patients with stage III or IV NSCLC between 2004 and 2011 and they highlighted that long-term survivors of stage IV with complete remission had primary tumors of small size and fewer metastases, which were considered independent prognostic factors for overall survival among long-term survivors.

Chelal et al. [24] reported in 2022 the case of a patient with stage IV non-small cell lung adenocarcinoma with complete response for 5 years post-first-line Nivolumab immunotherapy.

Quirynen et al. [25] reported in 2023 the case of a single patient with metastatic lung adenosquamous carcinoma with long-term and complete remission after IT, although the adverse reactions were severe (severe Pembrolizumab-induced immune-related encephalitis), and the treatment was interrupted.

Samanci et al. [26] reported in 2020 the case of a stage IV ALK-positive NSCLC patient with complete response over 3.5 years under third-line Ceritinib treatment. Alkassis et al. [27] reported in 2021 a long-term survival in a patient with EGFR mutation-positive NSCLC with no evidence of disease for eight years.

The therapeutic approach for the second-line treatment of patients with metastatic non-small cell lung cancer (NSCLC) without mutations has been revolutionized by the approval of immune checkpoint inhibitors (ICIs) (Nivolumab, Pembrolizumab, Atezolizumab), and the choice of the most suitable second-line treatment is a challenge. For cancers with squamous histology, Nivolumab, Pembrolizumab (in patients with PD-L1  >  1%) or Atezolizumab have proven to be more effective than chemotherapy [28,29,30,31].

Nivolumab and Atezolizumab are indicated as monotherapy for the treatment of bronchopulmonary cancer other than small cell lung cancer, locally advanced or metastatic, after previous chemotherapy treatment in adults.

Pembrolizumab is indicated as monotherapy for the treatment of NSCLC, locally advanced or metastatic, in adults whose tumors express PD-L1 with an STP ≥ 1% and who have previously received at least one chemotherapy regimen.

As we described in the case presentation, for our patient, we chose second-line immunotherapy with Nivolumab.

Guo et al., in a recent review, in 2024, highlight the advances and challenges of first-line immunotherapy for non-small cell lung cancer [32].

Carbone et al. [33], in a four-year clinical update published in 2024, outline the outcomes with first-line Nivolumab plus Ipilimumab with chemotherapy for metastatic non-small cell lung cancer.

Wang et al. [34] published the results of a phase 2 study in 2024. They highlight that Nivolumab combined with Docetaxel versus Nivolumab in patients with previously treated non-small cell lung cancer may be a promising treatment option for NSCLC patients after progression on first-line chemotherapy that is platinum-based.

Recent studies suggest that the combination of immunotherapy and radiation can increase the abscopal phenomenon, a systemic response seen when a primary tumor is irradiated or combined with immunotherapy, and the consequence is the stimulation of an immune response against tumors outside the radiation field at distant sites [35,36].

The abscopal effect has been seen especially in melanoma and NSCLC [37].

A review of possible biomarkers which could predict the abscopal effects, following radiotherapy or ICI initiation, highlighted that the presence of tumor-infiltrating lymphocytes in biopsy material or a higher neutrophil to lymphocyte ratio, before the initiation of the treatment, could have a predictive value [38].

The literature suggests that abscopal effects occur in highly immunogenic tumors: renal cell carcinoma (RCC), non-small cell lung cancer (NSCLC), head and neck squamous cell carcinoma, melanoma and hepatocellular carcinoma, but also in other histological subtypes [39,40,41].

The particularities of this case are outlined below: a young patient, with non-small lung cancer stage IV, with the onset of the disease through neurological symptoms determined by brain metastasis, the immunohistochemical result raised problems of differential diagnosis, the complete favorable response 20 months after the initiation of immunotherapy, maintaining this response 6 months later, maintaining ECOG = 0 performance status, very good psycho-emotional status, throughout the 3 years from the time of diagnosis.

## 4. Conclusions

Immunotherapy as a second-line treatment is beneficial to the survival of patients with metastatic NSCLC with disease progression beyond initial chemotherapy.

The abscopal effects of radiotherapy have attracted a lot of interest due to recent development and the use of immunotherapy.

The combination of radiotherapy and immunotherapy has the potential to play an important role in metastatic NSCLC.

## Data Availability

The raw data supporting the conclusions of this article will be made available by the authors on request.
